# Refining Uniform Discrimination Metrics: Towards a Case‐by‐Case Weighting Evaluation in Species Distribution Models With Presence‐Absence Data

**DOI:** 10.1002/ece3.72573

**Published:** 2025-12-11

**Authors:** Alberto Jiménez‐Valverde

**Affiliations:** ^1^ Department of Biogeography and Global Change Museo Nacional de Ciencias Naturales (MNCN), CSIC Madrid Spain

**Keywords:** ecological niche models, evaluation, presence‐absence models, representativeness effect, ROC analysis, sensitivity‐star

## Abstract

Species distribution models are widely used in ecological research, but their validation remains challenging due to the representative effect. This effect, which reflects the strong dependence of discrimination performance on the distribution of suitability values, hampers the comparison and generalization of discrimination statistics across datasets. This study aims to address this issue by refining uniform discrimination metrics (e.g., *uAUC* and uSe*) to better harmonize evaluation scores and allow for biological interpretation of model performance differences. I propose an alternative method for calculating uniform discrimination scores that directly incorporates weights, eliminating the need for the resampling procedure in the original formulation. Through simulations, I demonstrate that this approach reduces bias and improves the coverage of 95% confidence intervals. Furthermore, the method provides a pathway to account for uncertainty associated with presence‐absence data during model validation, offering a more robust evaluation framework. By addressing the representative effect and refining evaluation metrics, this study enhances the reliability of species distribution model validation. These improvements facilitate the meaningful comparison of model performance across datasets and support more accurate ecological interpretations.

## Graphical abstract

This study addresses the challenge of validating species distribution models by refining uniform discrimination metrics to mitigate the representative effect. By introducing a weighted calculation method that eliminates resampling, the approach reduces bias, improves confidence interval coverage, and accounts for uncertainty in presence‐absence data. These advancements enhance model evaluation reliability, facilitating meaningful comparisons and robust ecological insights.

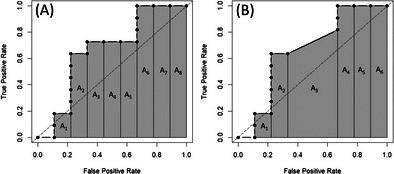

## 1 Introduction

Species distribution modeling (SDM) has become a dominant approach in the fields of ecology, biogeography, and conservation (Franklin 2009; Peterson et al. 2011). Typically, the main goal of an SDM exercise is to produce a probability of presence, or some sort of continuous habitat suitability score, that best classifies the instances of presence and absence of the focus species (Booth et al. 2014; Guisan et al. 2017) so that it can be used, for instance, to predict its distribution in new spatial or temporal scenarios. Before using the models, we must evaluate them, which usually means applying the metrics derived from the four cells of confusion matrices (true positive rate or sensitivity, true negative rate or specificity, false positive rate, and false negative rate; Fielding and Bell 1997). The area under the ROC curve (*AUC*) is a widely accepted metric in SDM (Lobo et al. 2008), although other measures such as sensitivity‐star (Se*; Jiménez‐Valverde 2014) have been proposed as interesting alternatives.

Changes in classification performance of a distribution model between datasets could be interpreted as changes in the response of the species to the ecogeographic factors (the predictors) considered (Jiménez‐Valverde 2022). For instance, in a certain territory, the species could be in environmental disequilibrium due to dispersal restrictions, or it is released from the biotic restrictions it suffers in other areas, or perhaps the predictors considered could no longer be as relevant as modeled. Thus, the distribution models might not work as one would ideally expect but still be useful for generating hypotheses about the determinants of a species' geographic range and for fostering further research. However, to ascribe a biological cause to a change in the performance of the distribution models, we must first account for the environmental representativeness effect; this is, the lack of comparability of discrimination performance due to dissimilarities in the distribution of the suitability values between datasets, which in turn depends on the distribution of the ecogeographic variables considered (Jiménez‐Valverde et al. 2013; Jiménez‐Valverde 2022; see also Lawson et al. 2014). Differences in discrimination performance are expected even if the species responds to the environment in the same way (Jiménez‐Valverde 2022). Thus, differences in discrimination capacity can be due to biological causes, to the representativeness effect or, in most if not all cases, to a combination of both factors. Consequently, comparative SDM studies aiming to generalize their findings solely based on the discriminative capacity of the models may lack the so desired comparability and broad applicability (Jiménez‐Valverde et al. 2013).

Recently, I proposed a method to account for the representativeness effect and showed how our interpretation of the distribution models can change significantly once we control for this effect (Jiménez‐Valverde 2022). The method involves calculating the so‐called uniform statistics using a stratified bootstrap approach with inverse probability weighting. This harmonizes the discrimination performance in different datasets and makes them comparable. In this contribution, I tested an alternative way to calculate the uniform *AUC* (*uAUC*) and uniform Se* (uSe*) that avoids resampling (the bootstrapping) by directly adding weights when calculating the true positive and false positive fractions along the ROC curve. I expected that the bias and precision of the estimated *uAUC* and uSe* scores would improve, and consequently so would the coverage of the confidence intervals (CIs). Additionally, incorporating weights into the evaluation procedure allows discrimination metrics to take into account the varying uncertainty in presence‐absence data quality, including issues like species misidentification, positional errors, and unreliable absence records.

First, I will describe how to incorporate case‐by‐case weights into ROC analysis. I will begin by outlining the trapezoidal method for calculating the discrimination statistics, then explain how weights can be straightforwardly included, and finally use a simple example to illustrate how weights affect the ROC curve and the derived statistics. Second, I will explain how the uniform discrimination statistics represent a special case of weighting and how the previous (Jiménez‐Valverde 2022) and new approaches to compute them differ. Third, I will run simulations to compare both approaches in terms of bias, precision, and coverage of the CIs. Finally, I will discuss the results and outline possibilities for further research.

## 2 Case Weighting in ROC Analysis

**FIGURE 1:**
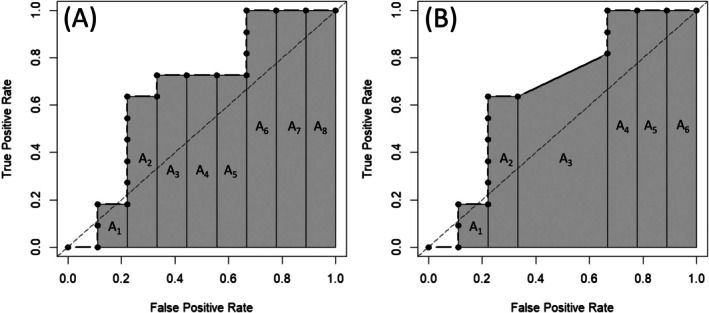
(A) Empirical ROC curve derived from a dataset without ties (data from Table 1), so the points of the curve are joined by vertical and horizontal lines forming a curve shaped like a staircase (Krzanowski and Hand 2009); the sum of the areas A_1_–A_8_ yields the *AUC* statistic. (B) Empirical ROC curve derived from a dataset with ties (the vector *hs*_*i*_ from Table 1 was modified so that the cases *i* = {11, …, 15} were assigned the same suitability score of 0.3), so now some points of the ROC curve are joined by diagonal lines; the sum of the areas A_1_–A_6_ yields the *AUC* statistic.

If the habitat suitability score is continuous and has enough resolution, then there is a zero probability of ties (instances of presence and absence with the same suitability values) being present, and the points of the empirical ROC curve (i.e., the true and false positives rate pairs for each threshold of the suitability score) are connected by vertical and horizontal lines (Krzanowski and Hand 2009; Nakas et al. 2023), as illustrated in Figure 1A. Although perhaps less common in SDM, ties can occur when the suitability score lacks sufficient resolution, for example, if the model output is ordinal (e.g., Stockwell 1999) or if continuous scores are categorized post hoc (e.g., Román Muñoz et al. 2005). In such cases, some ROC points are connected by diagonal lines (Krzanowski and Hand 2009; Nakas et al. 2023), as shown in Figure 1B. In both scenarios, the *AUC* can be estimated by the trapezoidal rule (Bamber 1975) as the sum of the areas of the rectangles (no ties) or trapezoids (with ties) formed between the ROC points (A_1_–A_8_ in Figure 1A and A_1_–A_6_ in Figure 1B). The *AUC* estimated in this manner is equivalent to the estimation obtained using the Mann–Whitney *U* statistic (Bamber 1975; Hanley and McNeil 1982), with both methods providing unbiased estimates of the underlying *AUC* if ties are not present (Zweig and Campbell 1993; Hajian‐Tilaki et al. 1997; Faraggi and Reiser 2002; Krzanowski and Hand 2009; Jiménez‐Valverde 2020). While in the absence of ties the estimated *AUC* can be interpreted as the probability that a presence chosen at random will be assigned a higher suitability score than a randomly chosen absence (Bamber 1975), this interpretation does not hold when ties are present (Krzanowski and Hand 2009; Zou et al. 2012). In any case, the trapezoidal rule is easily implemented in R (R Development Core Team 2024) as follows. Let **hs** be the vector of habitat suitability scores sorted in decreasing order, and **sp** the vector of associated instances of presence and absence of the species. All unique **hs** values are identified and, for each value (threshold), the cumulative true positive and false positive rates (*tp* and *fp*, respectively) are calculated by grouping observations with identical **hs** values and summing the number of cases in which **sp** = 1 and in which **sp** = 0, respectively. These rate pairs (*tp*, *fp*) are the points that define the ROC curve and are used to compute the *AUC* using the trapezoidal rule as:

$$\mathrm{sum}\left(\text{diff}{\left(\mathrm{fp}\right)}^{*}\left(\text{head}\left(\mathrm{tp},-1\right)+\text{tail}\left(\mathrm{tp},-1\right)\right)/2\right)$$

Case‐by‐case weighting can now be easily incorporated into ROC analysis, as follows. Being **w** the vector of weights associated to each case, for each threshold, the weighted true positive and false positive rates (*tp.w* and *fp.w*, respectively) are cumulatively updated by summing *w*_*i*_ of the presences and absences, respectively, with that score. The weighted *AUC* (*wAUC*) is calculated via the trapezoidal rule as before but substituting *tp* and *fp* by *tp.w* and *fp.w*, respectively. Weights can also be incorporated into the calculation of Se* (wSe*) by finding the values tp.w and (1—fp.w) that minimize their difference and then averaging them (Jiménez‐Valverde 2020). The new function AUCuniform.2() of the vandalico package version 0.2.0 (Jiménez‐Valverde 2025a) allows the calculation of *wAUC* and wSe* using this direct weighted trapezoidal estimation procedure.

The main point to keep in mind is that what matters is the relative value of the weights within each state of the binomial variable presence‐absence and, therefore, it is not a matter of weighting absences against presences (i.e., penalizing false positives less or more than false negatives; see Metz 1978). Imagine two vectors, **hs** and the associated **sp**, with twenty cases (*i* = {1, …, 20}) and a corresponding *AUC* value of 0.667, as in Table 1. If **w.a** is a vector of weights such that each element *w.a*_*i*_ is determined by the corresponding element *sp*_*i*_ as follows:

$$w.a_{i}=\text{1if}\;{\mathrm{sp}}_{i}=1$$

$$w.a_{i}=0.2\;\text{if}\;{\mathrm{sp}}_{i}=0,$$

then the *wAUC* does not differ (*wAUC* = 0.667, Table 1) and the ROC curves, weighted and unweighted, are identical (Figure 1A). Any other weighting values would produce the same results as long as they are constant within each group of presences and absences.

**TABLE 1:** Different weighting examples.

Case (*i*)	*hs*_*i*_	*sp*_*i*_	*w.a*_*i*_	*w.b*_*i*_	*w.c*_*i*_	*w.d*_*i*_	*w.e*_*i*_	*w.f*_*i*_
1	1.00	0	0.2	1	0.2	1	1	1
2	0.95	1	1	1	1	1	0.2	0.04
3	0.90	1	1	1	1	1	1	0.2
4	0.85	0	0.2	1	1	1	1	1
5	0.80	1	1	1	1	1	1	0.2
6	0.75	1	1	1	1	1	1	0.2
7	0.70	1	1	1	1	1	1	0.2
8	0.65	1	1	1	1	1	1	0.2
9	0.60	1	1	1	1	1	1	0.2
10	0.55	0	0.2	1	1	1	1	1
11	0.50	1	1	1	1	1	1	0.2
12	0.45	0	0.2	1	1	0.2	1	1
13	0.40	0	0.2	1	1	1	1	1
14	0.35	0	0.2	1	1	1	1	1
15	0.30	1	1	1	1	1	1	0.2
16	0.25	1	1	1	1	1	1	0.2
17	0.20	1	1	1	1	1	1	0.2
18	0.15	0	0.2	1	1	1	1	1
19	0.10	0	0.2	1	1	1	1	1
20	0.05	0	0.2	0.2	1	1	1	1
*AUC*	0.667							
*wAUC*			0.667	0.634	0.732	0.661	0.649	0.649

*Note:* *hs*_*i*_, the habitat suitability scores vector; *sp*_*i*_, the presence‐absence vector; *w.a*_*i*_, …, *w.f*_*i*_, the weighting vectors; *AUC*, the area under the ROC curve; *wAUC*, the weighted AUC. The ROC curve derived from both the unweighted data and weighting scenario **w.a** is shown in Figure 1A, and the ROC curves associated with the other five weighting scenarios are shown in Figure 2 (see text for details).

**FIGURE 2:**
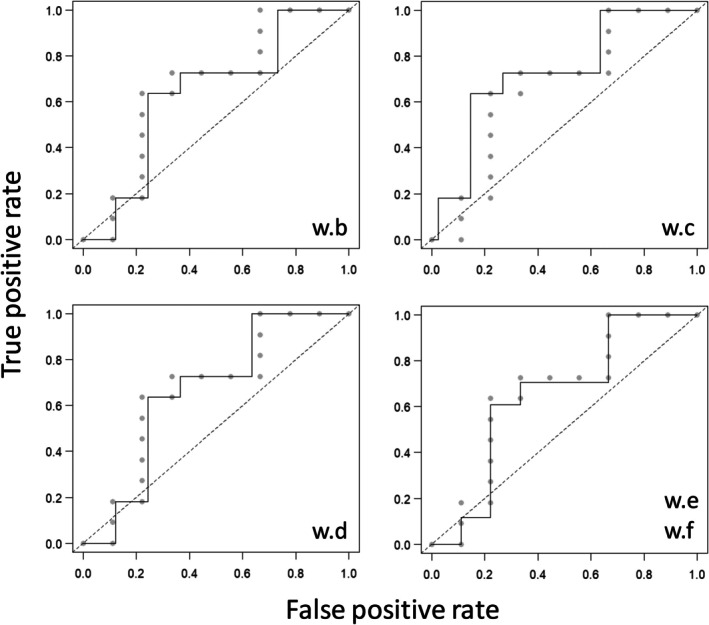
ROC curves corresponding with different weighting examples of Table 1. Gray dots, unweighted ROC curve; black line, weighted ROC curve. The ROC curve corresponding to the first weighting scenario **w.a** (see Table 1) is not shown since, as explained in the text, it is the same as the unweighted curve which is shown in Figure 1A.

If a low weight is assigned to an instance of absence associated with a low *hs*_*i*_ value, as it is the case with **w.b** (Table 1), then the *wAUC* will be lower than the *AUC*. On the contrary, if a low weight is assigned to an instance of absence associated with a high *hs*_*i*_ value, as in the case of **w.c** (Table 1), then the *wAUC* will be higher than the *AUC*. In both scenarios, the absolute difference between *wAUC* and *AUC* decreases as the cases depart from the initial and terminal points of the ordered **hs** vector (compare **w.b** with **w.d**). The inflection point at which |*wAUC*—*AUC*| equals zero (or is minimal in empirical curves) depends on the concrete distribution of the instances of presence and absence. If a low weight is assigned to a presence instead of an absence, then the pattern reverts, that is, if the instance of presence is associated with a high *hs*_*i*_ value, as in **w.e** (Table 1), then the *wAUC* will be lower than the *AUC*. Finally, note again that what matters are the relative values of the weights, not their absolute values; the vector **w.f** yields the same *wAUC* value as **w.e** (Table 1). The ROC curves associated with these five different weighting scenarios are shown in Figure 2.

## 3 The Uniform *AUC* and Uniform se*

The *uAUC* (and uSe*) is a special case of *wAUC* (and wSe*) aimed to account for the environmental representativeness effect (Jiménez‐Valverde 2022). The original proposal is based on a bootstrapping procedure with inverse probability weighting, so that **hs** is divided into a given number of bins and the weight of each case *i* is the inverse of the sample size of the corresponding bin. Then cases are resampled with replacement, the probability of each case being sampled equal to its weight. The uniform statistic is finally computed as the average of its bootstrapped values. The idea is to make the suitability scores follow a uniform distribution so that the discrimination statistics are harmonized and can be compared between datasets. The method is successful but data‐demanding and needs a sample size of 150 to achieve a negligible bias (Jiménez‐Valverde 2022). Also, the coverage of the 95% CIs seems to depend on sample size and on the concrete original distribution of the suitability values and even though the percentile method works reasonably well for the *uAUC*, coverage dramatically departs from the nominal value in the case of uSe* (Jiménez‐Valverde 2022).

The direct weighted trapezoidal estimation procedure bypasses the need for resampling and averaging, providing a direct calculation of the uniform (weighted) statistics. Following the original approach, the dataset is partitioned into bins, and each case *i* is assigned a weight equal to one divided by the number of observations in the corresponding bin. The *uAUC* and uSe* are then calculated as described in the previous section, which may improve their estimation (by decreasing bias and increasing precision) and the CIs (by improving coverage). To test this hypothesis, I replicated the simulations performed in Jiménez‐Valverde (2022) to compare both approaches.

## 4 Simulation Methods and Results

**FIGURE 3:**
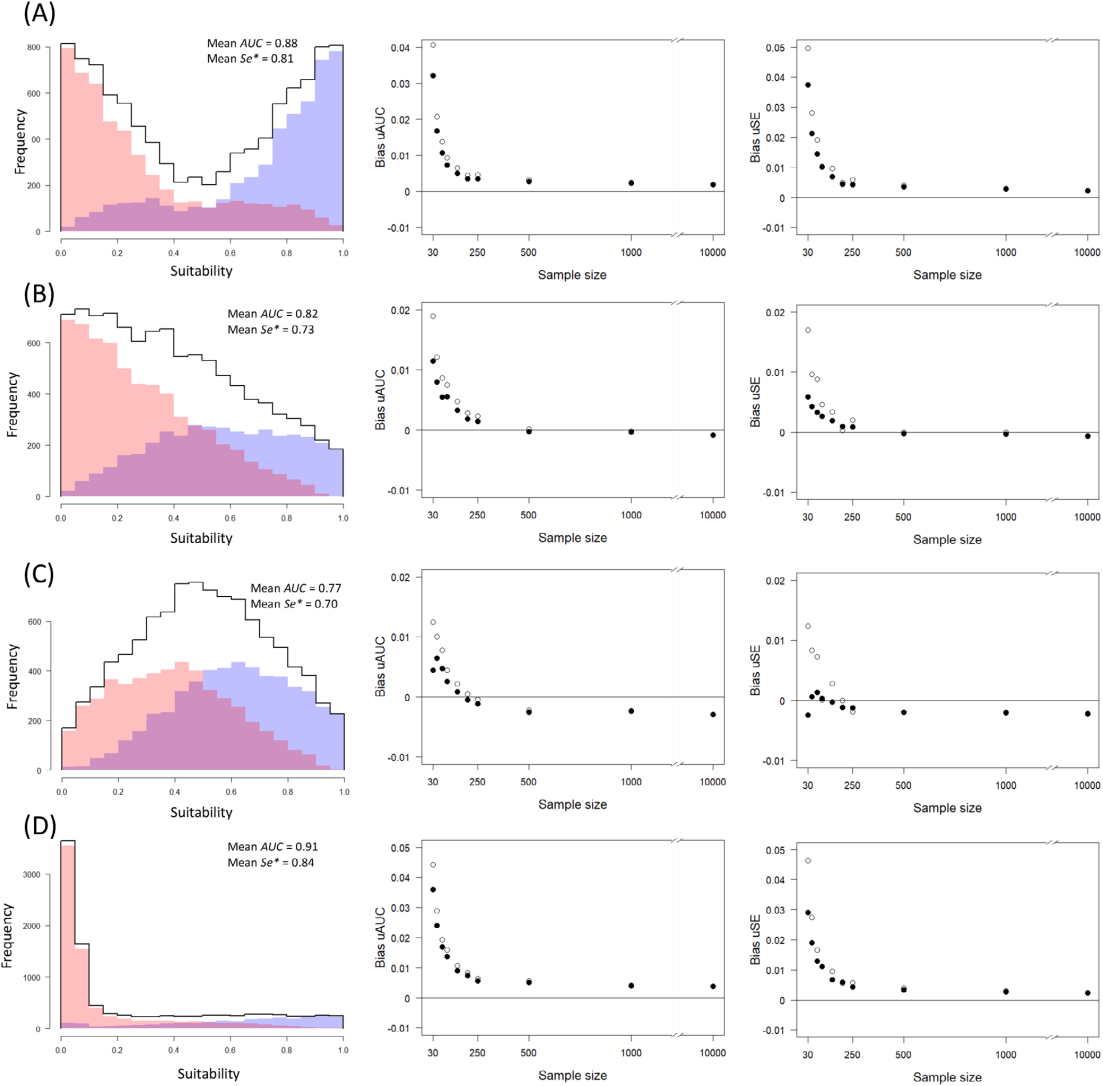
Examples of distributions of suitability values for the four simulated scenarios (A, B, C, and D) are depicted in the first column: Overall suitability (**hs**, black line), suitability values for presences (blue), and suitability values for absences (red). These examples correspond to one simulation for a sample size of 10,000. (A) Scenario A represents situations where low and high suitability values are more frequent than cases with medium values. (B) Scenario B represents situations in which the frequency of cases continuously decreased from low to high suitability values. (C) Scenario C represents situations where medium suitability values are more frequent than cases with low and high suitability values. (D) Scenario D represents situations where high and medium suitability values are less frequent than cases with low values. Mean *AUC* and Se* values—the average of the 10,000 iterations for *n* = 10,000—are indicated. Bias of the *uAUC* and uSe*, for each scenario and sample size, are depicted in the second and third columns, respectively. White dots, statistics calculated with the original bootstrapping method (Jiménez‐Valverde 2022); black dots, statistics calculated with the direct weighted trapezoidal estimation method.

I simulated four scenarios corresponding to different distributions of **hs** (scenarios A, B, C, and D; see Figure 3) and I considered 10 sample sizes (*n* = {30, 50, 74, 100, 150, 200, 250, 500, 1000, 10,000}). For scenario A, *n*/2 random numbers were generated from two truncated normal distributions bounded between 0 and 1 and with *μ*_1_ = 0, *μ*_2_ = 1 and *σ*_1_ = *σ*_2_ = 0.25, and concatenated. For scenario B, *n* random numbers were generated from a truncated normal distribution bounded between 0 and 1 and with *μ* = 0 and *σ* = 0.6. For scenario C, *n* random numbers were generated from a truncated normal distribution bounded between 0 and 1 and with *μ* = 0.5 and *σ* = 0.3. For scenario D, *n*/2 random numbers were generated from a truncated normal (with *μ* = 0 and *σ* = 0.05) and from a uniform distribution, both bounded between 0 and 1, and concatenated. For each scenario and sample size, I ran 10,000 iterations. In each iteration, first, a vector *hs*_*i*_ was simulated. Second, to simulate perfectly calibrated models, a vector *rnd*_*i*_ was generated by picking a sample of *n* random numbers from a uniform distribution. Third, to create the vector *sp*_*i*_ (presence or absence of the species), the condition:

$$\text{if}\;{\mathrm{rnd}}_{i}<{\mathrm{hs}}_{i}\;\text{then}\;{\mathrm{sp}}_{i}=1,\text{else}\;{\mathrm{sp}}_{i}=0$$

was set. Fourth, the *AUC* was estimated via the Mann–Whitney *U* statistic (Bamber 1975), and Se* was calculated by selecting the point that minimized the absolute difference between sensitivity and specificity, and by getting the mean of those values (Jiménez‐Valverde 2020). Finally, the *uAUC* and uSe* were calculated in two ways: via stratified bootstrapping with an inverse probability weighting (following Jiménez‐Valverde 2022) and via the direct weighted trapezoidal estimation procedure as explained above. For the calculation of the weights, 10 bins were used in both cases. For each scenario, the mean values for the *AUC* and Se* were calculated for *n* = 10,000 (see Figure 3). For each scenario, sample size and uniform metric, bias was computed as the difference between the median and the reference values of the uniform metric, whereas the interquartile range (IQR) was used as a measure of precision. Given that all the scenarios I simulated were perfectly calibrated, the reference values were always 0.83 for *uAUC* and 0.75 for uSe* (see Jiménez‐Valverde 2022 and Jiménez‐Valverde et al. 2013 for derivations). Finally, to study the coverage (the probability of including the reference discrimination metric value: 0.83 for the *uAUC* and 0.75 for uSe*) of the 95% CIs estimated via the percentile method (Efron and Tibshirani 1993), I run 100 iterations for each **hs** distribution detailed above and for *n* = {30, 500, 10,000} (as in Jiménez‐Valverde 2022). In each iteration, **sp** was generated as explained before, the data were resampled with replacement 1000 times (Hesterberg 2015), and the *uAUC* and uSe* were estimated via the direct weighted trapezoidal estimation method. CIs generated for both statistics were compared with those reported by Jiménez‐Valverde (2022, see table 1). The truncnorm (Mersmann et al. 2018) and vandalico packages for R were used to perform the analyses, which can be reproduced using Jiménez‐Valverde (2025b).

**FIGURE 4:**
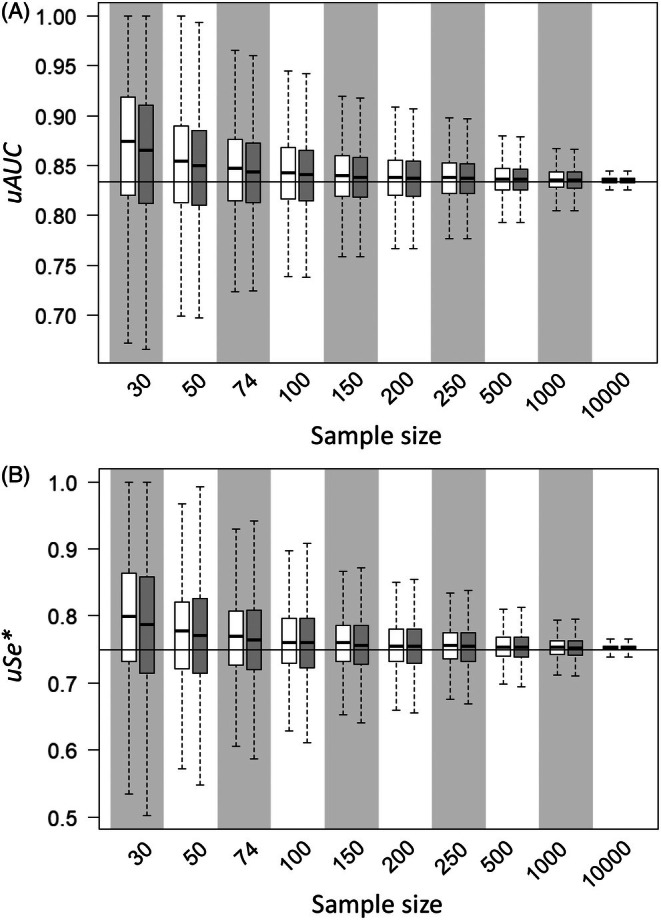
Median (horizontal marks), interquartile range (boxes), and 1.5 × spread (whiskers) of the (A) *uAUC* and (B) uSe* calculated using the original bootstrapping method (Jiménez‐Valverde 2022) (white boxplots) and the direct weighted trapezoidal estimation method (gray boxplots). Results are shown for each sample size of scenario A (see Figure S1 for the other three scenarios). The horizontal line marks the reference values (0.83 for the *uAUC* and 0.75 for uSe*).

As with the *uAUC* and uSe*obtained via bootstrapping, the direct weighted trapezoidal estimation method successfully harmonized the two discrimination measures, eliminating the differences between scenarios (Figure 4 and Figure S1) and converging on average to the reference values of 0.83 and 0.75 in all four cases. Whereas precision neither improved nor worsened with the direct weighted trapezoidal estimation method (Figure S2), bias slightly decreased, especially at the lowest sample sizes (Figure 3). It is worth noting that the distribution of **hs** influenced the direction of the bias, which was generally positive but could also be negative. In any case, bias was consistently low for both methods, remaining below |0.05|. Regarding the 95% CIs, in the case of the *uAUC*, there was not an improvement, and the pattern remained the same for the two methods; coverage was around the nominal value of 0.95 but extreme scenarios (A and D) are more challenging, especially at very high and very low sample sizes (Table 2). On the contrary, coverage significantly improved for uSe*, being around 0.95 in all scenarios and sample sizes (Table 2).

**TABLE 2:** Coverages of the 95% confidence intervals calculated with the percentile method for the *uAUC* and uSe* estimated via the direct weighted trapezoidal estimation method.

Scenario	*n* = 30	*n* = 500	*n* = 10,000
*uAUC*			
A	0.84 (0.92)	0.91 (0.96)	0.70 (0.75)
B	0.93 (0.95)	0.90 (0.92)	0.92 (0.86)
C	0.91 (0.89)	0.94 (0.92)	0.94 (0.90)
D	0.73 (0.80)	0.95 (0.83)	0.69 (0.55)
uSe*			
A	0.92 (1)	**0.97 (0.03)**	**0.96 (0)**
B	0.93 (0.93)	**0.93 (0.01)**	**0.96 (0)**
C	0.96 (0.84)	**0.97 (0.01)**	**0.88 (0)**
D	0.86 (0.91)	**0.92 (0.07)**	**0.93 (0)**

*Note:* In parentheses, results for the *uAUC* and uSe* estimated with the bootstrapping method originally proposed in Jiménez‐Valverde (2022, extracted from table 1). The scenarios in which the coverage noticeably improved with the new method are marked in bold.

## 5 Conclusions

The SDM field has grown at such an exponential rate (Lobo et al. 2010) that methods are being adopted much faster than they can be critically assessed. The lack of comparability of results due to methodological subtleties has been raised on several occasions. One example is the fact that species prevalence biases presence probabilities, so models for different species are hardly comparable in terms of suitability, and rescaling is usually required (Jiménez‐Valverde and Lobo 2006; Real et al. 2006). Another example, the one that concerns us in this contribution, is that of the representativeness effect hampers the comparison and generalization of evaluation metrics since the distribution of the habitat suitability scores conditions the value of the discrimination metrics (Jiménez‐Valverde et al. 2013). The harmonization of these metrics was suggested in Jiménez‐Valverde (2022) with the implementation of a stratified bootstrap procedure with inverse probability weighting. Here, I propose to simplify the methodology by avoiding resampling and directly adding weights in the trapezoidal estimation procedure, which successfully reduces bias at low sample sizes, both for the *uAUC* and uSe*. Also, the CIs for the uSe* statistic show much better coverage than with the original method. Disparities in discrimination metrics simply indicate better performance within one dataset compared to another; to add value to presence‐absence modeling studies, uniform statistics should always be reported for the sake of generalization and comparability (Jiménez‐Valverde 2022).

In this contribution I have focused on a particular case of weighted metrics, the so‐called uniform statistics. Yet, the consideration of weights in the evaluation procedure opens the possibility to directly include uncertainty associated with the quality of the data in the estimation of the discrimination metrics. A record of a species is usually strong proof of presence, but misidentifications are certainly not uncommon, especially in groups that suffer from an uncertain taxonomy (e.g., Coca‐de‐la‐Iglesia et al. 2024). Positional errors are also a common source of uncertainty associated with the quality of the instances of presence (Gábor et al. 2023). More questionable is the reliability of the instances of absence since they can be simply due to a lack of enough survey effort so that the species has gone undetected (Lobo et al. 2010). Different criteria can be used to weight both the instances of presence and absence according to their reliability like, for instance, the degree of expertise of the person who reported the record in the case of the presences (e.g., Tessarolo et al. 2021) or the degree of completeness of the biological inventories of the localities in the case of the absences (e.g., Lobo et al. 2018). The calculation of weighted evaluation statistics according to the uncertainty of the biological data is an issue that certainly deserves much further exploration in SDM.

## Author Contributions

**Alberto Jiménez‐Valverde:** conceptualization (lead), data curation (lead), formal analysis (lead), funding acquisition (lead), investigation (lead), methodology (lead), project administration (lead), resources (lead), software (lead), supervision (lead), validation (lead), visualization (lead), writing – original draft (lead), writing – review and editing (lead).

## Funding

This work was supported by Ministerio de Ciencia, Innovación y Universidades/Agencia Estatal de Investigación/FEDER, PID2023‐147672NB‐I00. Consejo Superior de Investigaciones Científicas, 2024ICT253.

## Ethics Statement

The author has nothing to report.

## Consent

The author has nothing to report.

## Conflicts of Interest

The author declares no conflicts of interest.

## Supporting information


**Appendix S1:** ece372573‐sup‐0001‐Supinfo.docx.

## Data Availability

No new data were used or generated. The code used for the analyses is available at Figshare (https://doi.org/10.6084/m9.figshare.28202207.v2).
