# Supplementary material for: Refining Uniform Discrimination Metrics: Towards a Case‐by‐Case Weighting Evaluation in Species Distribution Models With Presence‐Absence Data
Source: Ecol Evol. 2025 Dec 11;15(12):e72573. doi: 10.1002/ece3.72573 (PMC12698938; doi:10.1002/ece3.72573)
Supplement: Supplementary file 1 — Appendix S1: ece372573‐sup‐0001‐Supinfo.docx. [file ECE3-15-e72573-s001.docx]

**Refining uniform discrimination metrics: towards a case-by-case weighting evaluation in species distribution models with presence-absence data**

**Figure S1.**- Median (horizontal marks), interquartile range (boxes), and 1.5 × spread (whiskers) of the *uAUC* (left column) and *uSe** (right column) calculated using the original bootstrapping method (Jiménez-Valverde, 2022) (white boxplots) and the direct weighted trapezoidal estimation method (gray boxplots). Results are shown for each sample size of (A) scenario B, (B) scenario C and (C) scenario D. Horizontal line marks the reference values (0.83 for the *uAUC* and 0.75 for *uSe**).

**Figure S2.**- Precision (IQR, interquartile range) of the *uAUC* (left column) and *uSe** (right column) for each scenario (rows) and sample size. White dots, statistics calculated with the original bootstrapping method (Jiménez-Valverde, 2022); black dots, statistics calculated with the direct weighted trapezoidal estimation method.
